# A Predictive Transcriptomic Approach to the Resveratrol-Mediated Reversal of Hypothalamic Alterations in a Mouse Model of Obesity

**DOI:** 10.3390/genes17030297

**Published:** 2026-02-28

**Authors:** Brenda De la Cruz-Concepción, Juan Miguel Mendoza-Bello, Fredy Omar Beltrán-Anaya, Mónica Ramírez, Yaccil Adilene Flores-Cortez, Gema Damian-Sánchez, Eugenia Flores-Alfaro, Isela Parra-Rojas, Oscar Del Moral-Hernández, Miguel Cruz, Mónica Espinoza-Rojo

**Affiliations:** 1 Laboratorio de Biología Molecular y Genómica, Facultad de Ciencias Químico-Biológicas, Universidad Autónoma de Guerrero, Av. Lázaro Cárdenas S/N, Ciudad Universitaria, Chilpancingo 39086, Guerrero, Mexico; brenddlcruzc@gmail.com (B.D.l.C.-C.); jmiguelmb29@gmail.com (J.M.M.-B.); adilene_cortez01@hotmail.com (Y.A.F.-C.); gemadamiansanchez@gmail.com (G.D.-S.); 2 Laboratorio de Diagnóstico e Investigación en Salud, Facultad de Ciencias Químico-Biológicas, Universidad Autónoma de Guerrero, Av. Lázaro Cárdenas S/N, Ciudad Universitaria, Chilpancingo 39086, Guerrero, Mexico; frebeltran@hotmail.com (F.O.B.-A.); odelmoralh@gmail.com (O.D.M.-H.); 3 Laboratorio de Proteómica y Genómica Funcional, Facultad de Ciencias Químico-Biológicas, Universidad Autónoma de Guerrero, Av. Lázaro Cárdenas S/N, Ciudad Universitaria, Chilpancingo 39086, Guerrero, Mexico; 4 Laboratorio de Epidemiología Clínica y Molecular, Facultad de Ciencias Químico-Biológicas, Universidad Autónoma de Guerrero, Av. Lázaro Cárdenas S/N, Ciudad Universitaria, Chilpancingo 39086, Guerrero, Mexico; eugeniaflores@uagro.mx; 5 Laboratorio de Investigación en Obesidad y Diabetes, Facultad de Ciencias Químico-Biológicas, Universidad Autónoma de Guerrero, Av. Lázaro Cárdenas S/N, Ciudad Universitaria, Chilpancingo 39086, Guerrero, Mexico; iselaparra@uagro.mx; 6 Unidad de Investigación Médica en Bioquímica, Hospital de Especialidades, Centro Médico Nacional Siglo XXI, Instituto Mexicano del Seguro Social, Av. Cuauhtémoc 330, Colonia Doctores, Ciudad de Mexico 06720, Mexico

**Keywords:** obesity, hypothalamus, transcriptomic analysis, resveratrol

## Abstract

**Background:** Obesity is associated with hypothalamic dysfunction characterized by neuroinflammation and altered transcriptional programs. While resveratrol (RSV) has shown beneficial metabolic effects in peripheral tissues, its central effects on hypothalamic gene expression in obesity remain poorly understood. This study provides the first predictive transcriptomic analysis of the hypothalamic response to RSV in a mouse model of diet-induced obesity. C57BL/6 male mice were fed a high-fat diet (HFD) to induce obesity and then subsequently treated with RSV. **Methods:** Hypothalamic RNA was extracted and analyzed using RNA sequencing. Differentially expressed genes (DEGs) were identified and functionally analyzed through KEGG pathway analysis. **Results:** Although RSV did not significantly alter body weight, it reversed the expression of several HFD-induced DEGs. Key genes modulated by RSV included *Aqp7*, *Ccl27a*, *Lta*, *Rilp*, *M6pr-ps*, *C1ra*, *Snail1*, *Gbgt1*, and *Ppargc1b*, which are involved in inflammation, lipid metabolism, mitochondrial function, and immune signaling. Pathway enrichment analysis revealed significant modulation of TNF and NF-κB signaling, cytokine–cytokine receptor interactions, glycosphingolipid biosynthesis, and phagosome-related activity. Remarkably, 45% of RSV-responsive transcripts were non-coding RNAs, suggesting epigenetic regulation. **Conclusions:** RSV reprograms the hypothalamic transcriptome in obesity, targeting both coding and non-coding RNAs associated with inflammation and metabolic regulation, independently of weight loss. These findings identify RSV as a potential central modulator of metabolic dysfunction and highlight the hypothalamus as a promising therapeutic target in obesity-related disease.

## 1. Introduction

Obesity represents a growing global pandemic and is currently acknowledged as one of the primary risk factors affecting human health. It is defined as an excessive accumulation of lipids due to an imbalance between energy intake and expenditure [1]. Obesity caused by the consumption of a high-fat diet (HFD) is characterized by increased circulating levels of lipopolysaccharides [2] and pro-inflammatory cytokines, such as interleukin-6 (IL-6) and tumor necrosis factor-alpha (TNF-α), which trigger low-grade chronic inflammation in peripheral organs [3] and in the central nervous system (CNS) [2,4].

In the CNS, an HFD disrupts neuronal communication, impairs the function of the blood–brain barrier (BBB) [5], and promotes the recruitment of peripheral immune cells, facilitating the entry of pro-inflammatory mediators into the brain and activating microglia [6]. These effects are particularly evident in the hypothalamus, which is located near the median eminence, a region where the BBB is more permeable and maintains close communication with peripheral signals [2].

Within the hypothalamus, diet-induced obesity alters nutrient-sensing mechanisms, feeding behavior regulation, and energy homeostasis [4]. These alterations include disrupted insulin and leptin signaling hormones secreted by the pancreas and adipose tissue, respectively, in response to glucose levels and increased adipocyte energy stores [7]. These hormones bind to their receptors (IR and LepR, respectively) on first-order neurons in the hypothalamus [8], activating the phosphoinositide 3-kinase (PI3K)/protein kinase B (AKT) and the Janus kinase (JAK)/signal transducer and activator of transcription 3 (STAT3) pathways [8,9]. These signaling cascades regulate the expression of anorexigenic genes (e.g., *Proopiomelanocortin* (*Pomc*) and *Cocaine- and amphetamine-regulated transcript* (*Cart*)) [10], as well as orexigenic genes (e.g., *Neuropeptide Y* (*Npy*) and *Agouti-related protein* (*Agrp*)) [9,10,11].

Chronic HFD consumption in mice promotes systemic metabolic dysfunction and alters the hypothalamic expression of genes involved in food intake regulation, thereby stimulating appetite and increasing caloric intake [12,13,14], ultimately contributing to obesity.

In addition to protein-coding genes, growing evidence indicates that non-coding RNAs, particularly long non-coding RNAs (lncRNAs) and antisense transcripts, play a critical role in the regulation of hypothalamic inflammation and metabolic homeostasis. lncRNAs modulate gene expression through transcriptional, post-transcriptional, and epigenetic mechanisms, influencing metabolic and inflammatory signaling pathways and responding to nutritional and hormonal cues in key metabolic tissues [15,16]. Similarly, antisense RNAs fine-tune gene expression by regulating mRNA stability, transcriptional interference, and chromatin modifications, contributing to the control of metabolic and immune-related genes [17,18]. Dysregulation of these non-coding regulatory layers has been increasingly implicated in chronic inflammation and metabolic dysfunction associated with obesity, highlighting their potential relevance as mediators of central metabolic regulation [15,16,19].

In addition to HFD-induced hypothalamic neuroinflammation, obesity triggers increased production of reactive oxygen species and reduced antioxidant defenses, resulting in oxidative stress [4]. Oxidative stress contributes to insulin and leptin resistance and alters the function of effector molecules, including the aforementioned neuropeptides, as well as genes involved in inflammation [14]. Therefore, both neuroinflammation and oxidative stress play a critical role in gene expression changes that disrupt hypothalamic function and promote the persistence of obesity.

Natural compounds with antioxidant properties have shown promise in counteracting oxidative stress [20,21] and have demonstrated beneficial effects in obesity management [22]. Among these, resveratrol (RSV) [13,23,24,25], a polyphenolic compound abundant in grapes, berries, and red wine [26], has attracted considerable attention for its anti-inflammatory, antioxidant, neuroprotective, and anti-obesogenic properties [13,23,24,25].

RSV has been shown to confer neuroprotection through several mechanisms, many of which are associated with its antioxidant and anti-inflammatory actions [27,28,29]. In HFD-fed murine models with obesity, RSV supplementation protects against neuroinflammation, pyroptosis, and neuronal damage [28]; modulates proteolytic activity [27]; reduces neuronal apoptosis; and promotes hypothalamic neurogenesis toward anorexigenic POMC neurons [30].

In addition, RSV modulates the expression of various genes disrupted in obesity, including *Sirtuin 1* (*Sirt1*) [28], *Toll-like receptor 4* (*Tlr4*), *Tumor necrosis factor* (*Tnf*), *Interleukin 1β* (*Il1b*), *Il6* [31,32,33], *Il10* [26], *Nuclear factor kappa-B* (*Nfkb*), *C-C motif chemokine ligand 2* (*Ccl2*) [33,34], and *C-C chemokine receptor 2* (*Ccr2*) [34]. RSV also influences the expression of metabolic genes such as *Peroxisome proliferator-activated receptor delta* (*Ppard*), *gamma* (*Pparg*), and *ATP citrate lyase* (*Acly*) [33,34,35], as well as *Leptin* (*Lep*), *Leptin receptor* (*Lepr*), *Suppressor of cytokine signaling 3* (*Socs3*) [36] and *Insulin receptor substrate 1* (*Irs1*) [34].

Together, these findings support the hypothesis that antioxidant interventions may mitigate the transcriptomic alterations associated with diet-induced obesity.

The aim of this study was to characterize the transcriptomic alterations in the hypothalamus of HFD-induced mice with obesity, and to determine whether RSV supplementation modulates these changes in gene expression. To address this, we applied a predictive transcriptomic and bioinformatic approach to identify DEGs and the signaling pathways through which RSV may exert therapeutic effects in obesity. Unlike conventional descriptive RNA-seq studies, which primarily report differential gene expression profiles [37], this integrative strategy combines differential expression analysis with functional enrichment and pathway-based interpretation [38,39,40] to identify the coordinated gene networks and signaling pathways potentially targeted by resveratrol. This approach enabled the identification of candidate genes and signaling pathways with hypothalamic dysfunction, providing new insights into the central actions of RSV in obesity.

## 2. Materials and Methods

The feed for the standard diet (LabDiet 5001, St. Louis, MO, USA) and high-fat diet (HFD; Research Diets D12492, New Brunswick, NJ, USA) was obtained from commercial suppliers. Resveratrol (RSV; CAS No. 501-36-0) was purchased from Sigma-Aldrich (St. Louis, MO, USA), and TRIzol reagent (CAS No. 15596018) was obtained from Invitrogen (Carlsbad, CA, USA). All reagents used in this study were of analytical grade and confirmed to be of high purity.

### 2.1. Experimental Model of Obesity and RSV Treatment

Male C57BL/6 mice, 8 weeks old (n = 5 per group), were obtained from the Biotherium of the Faculty of Chemical-Biological Sciences, Autonomous University of Guerrero. All procedures involving animals were conducted in accordance with the Mexican guidelines (NOM-062-ZOO-1999) for the care and use of laboratory animals and approved by the Institutional Animal Care and Use Committee (CICUAL-01/2019) of the Autonomous University of Guerrero.

Mice were housed under controlled conditions (22 ± 2 °C; 50–60% humidity; 12 h light/12 h dark cycle) with ad libitum access to food and water. Animals were randomly assigned to three experimental groups:

1. Control (C), fed a standard diet (LabDiet 5001, St. Louis, MO, USA).
2. Obesity (Ob), fed a high-fat diet (HFD; D12492, Research Diets, New Brunswick, NJ, USA).
3. Obesity treated with RSV (Ob + RSV), fed the same HFD and treated with 2.25 mg/kg body weight of resveratrol (RSV; Sigma-Aldrich, CAS No. 501-36-0, St. Louis, MO, USA).

Each group consisted of five animals for body weight measurements, and three samples of the animals were sent for RNA-seq analysis. The HFD regimen was initiated at week 8 in the Ob and Ob + RSV groups, while the C group remained on a standard diet. Beginning at week 16, RSV was administered daily for 4 weeks by oral gavage (diluted in saline solution) at a dose of 2.25 mg/kg/day using an esophageal cannula. The RSV dose was selected based on previously reported studies demonstrating the metabolic effects of this antioxidant in HFD-fed murine models [41].

The C and Ob groups received an equivalent volume of saline. Body weight was recorded weekly throughout the study.

### 2.2. Tissue Collection and Hypothalamus Dissection

At the end of the treatment period, animals were anesthetized with sodium pentobarbital (126 mg/kg; Cheminova, Mexico City, Mexico) and euthanized. The brain was rapidly removed, and the hypothalamus was dissected and washed with phosphate-buffered saline (pH 7.4). Tissue samples were immediately processed or stored at −80 °C until RNA extraction.

### 2.3. Total RNA Extraction and Quality Assessment

Total RNA was extracted from the hypothalamus using TRIzol reagent (Invitrogen, CAS No. 15596018; Carlsbad, CA, USA), following the manufacturer’s instructions. Tissue homogenization was performed using a PRO 200 homogenizer (PRO Scientific Inc., Oxford, CT, USA).

RNA purity and concentration were assessed using a NanoDrop 2000c spectrophotometer (Thermo Fisher Scientific, Waltham, MA, USA), considering samples with A260/A280 ratios ≥ 1.9 and concentrations ≥ 20 ng/μL as acceptable. All RNA samples were stored at −80 °C until sequencing.

### 2.4. RNA Sequencing and Bioinformatic Analysis

mRNA sequencing was performed by Novogene Co., Ltd. (Sacramento, CA, USA). Quality control metrics included Q20, Q30 scores, and GC content. Clean reads were aligned to the reference mouse genome using HISAT2 (v2.0.5). Gene-level quantification was performed using featureCounts (v1.5.0-p3). Transcript abundance was normalized as fragments per kilobase of transcript per million mapped reads (FPKM).

### 2.5. Differential Gene Expression Analysis

Differential expression analysis was conducted using the DESeq2 R package (v1.20.0) with three biological replicates per group. *p*-values were adjusted using the Benjamini–Hochberg procedure [42] to control the false discovery rate (FDR). Genes with adjusted *p* < 0.05 and log_2_ FC ≥ 1 were considered DEGs.

### 2.6. Functional Enrichment Analysis

DEGs were annotated using the clusterProfiler R package (v3.8.1), which enabled the identification of gene families and their biological functions in the mouse genome based on public databases. Gene ontology (GO) enrichment analysis was conducted to determine the biological significance of each DEG in terms of biological process, molecular function, and cellular component. Additionally, statistical enrichment analysis of DEG was performed using the Kyoto encyclopedia of Genes and Genomes (KEGG) to identify associated signaling pathways. GO terms and KEGG pathways with an adjusted *p* < 0.05 and |log_2_ fold change| ≥ 1. were considered significantly enriched.

### 2.7. Statistical Analysis

Body weight data were analyzed using two-way ANOVA followed by Tukey’s multiple comparisons test. Results are presented as mean ± standard deviation (SD). Statistical analyses were performed using GraphPad Prism (v9.3.1), and *p* < 0.05 was considered statistically significant.

## 3. Results

### 3.1. Effect of RSV on Body Weight in an Obesity Model

**Figure 1:**
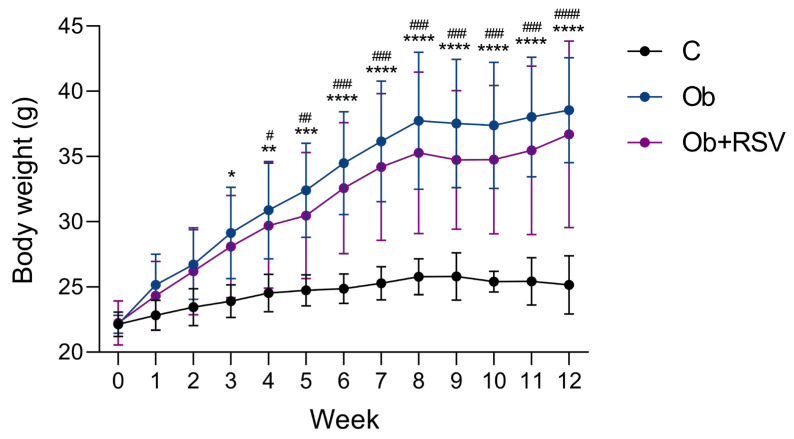
Effect of RSV on body weight. Body weight was compared among the experimental groups: control (C), obesity (Ob), and obesity treated with resveratrol (Ob + RSV). Significant differences in body weight were observed among all groups starting from week 2 of feeding with either the high-fat diet (HFD) or standard diet (C). Data are presented as mean ± SD and were analyzed using two-way ANOVA followed by Tukey’s multiple comparisons test. * *p* < 0.05 Ob vs. C, ** *p* < 0.01 Ob vs. C, *** *p* < 0.001 Ob vs. C, **** *p* < 0.0001 Ob vs. C. ^#^ *p* < 0.05 Ob + RSV vs. C, ^##^ *p* < 0.01 Ob + RSV vs. C; ^###^ *p* < 0.001 Ob + RSV vs. C, ^####^ *p* < 0.0001 Ob + RSV vs. C.

To induce obesity, mice were fed an HFD for eight weeks. One group remained with obesity (Ob), while the other received RSV treatment (Ob + RSV) during the final four weeks of the HFD regimen. A control group (C) was maintained on a standard diet. A significant increase in body weight was observed in both groups with obesity (Ob and Ob + RSV) compared with the control group, beginning at week 4 and persisting through week 12 (*p* < 0.05; Figure 1). However, no significant difference in body weight gain was detected between the Ob and Ob + RSV groups, indicating that RSV did not significantly affect weight gain under the experimental conditions.

### 3.2. Global Transcriptomic Analysis of Differentially Expressed Genes in the Hypothalamus

To investigate the global gene expression landscape, RNA-seq analysis was performed. Prior to differential gene expression analysis, the constructed libraries were subjected to quality control, ensuring their suitability (Supplementary Materials).

To identify hypothalamic DEGs associated with obesity and those modulated by RSV, gene-level counts were estimated for the three study groups based on gene length and the number of reads assigned to each gene. Differential expression analysis was performed using DESeq2, with DEGs defined by an adjusted *p* < 0.05 and |log_2_ fold change| ≥ 1.

**Figure 2:**
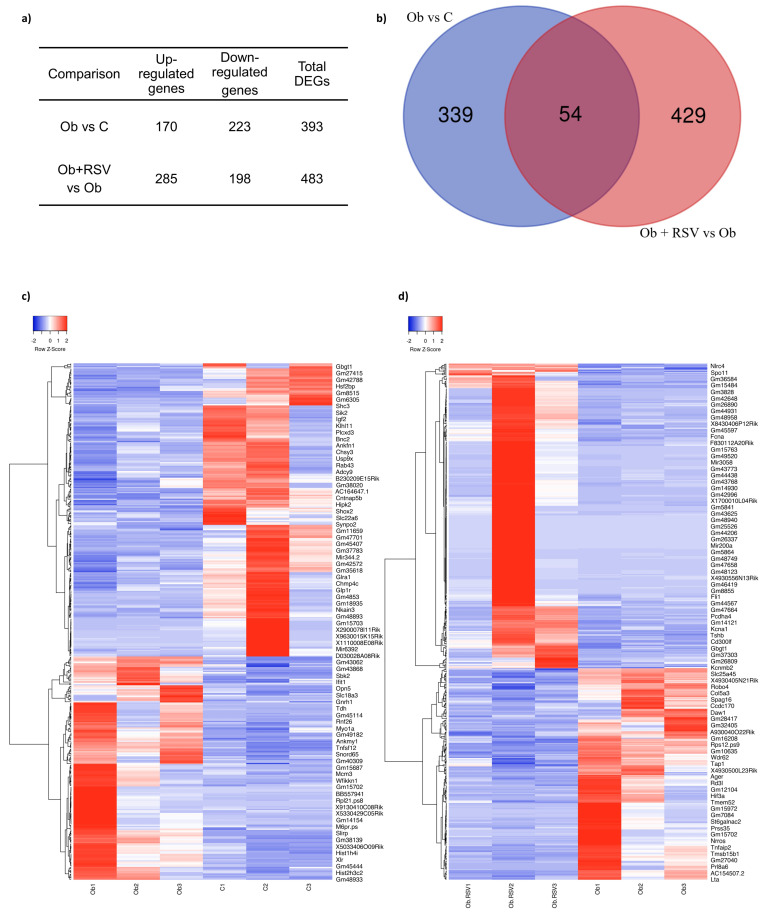
Differential gene expression in the hypothalamus. (**a**) Number of upregulated and downregulated genes identified in the different mouse models. (**b**) Venn diagram showing differentially expressed genes (DEGs), including 54 overlapping genes identified under obesity conditions and modulated by resveratrol treatment. (**c**) Heatmap of DEGs in the Ob vs. C comparison. (**d**) Heatmap of DEGs in the Ob + RSV vs. Ob comparison. Red indicates high expression levels; blue indicates low expression levels (adjusted *p* < 0.05 and log_2_ fold change ≥ 1).

RNA-seq analysis of hypothalamic tissue revealed 393 DEGs in Ob vs. C and 483 DEGs in Ob + RSV vs. C (adjusted *p* < 0.05 and log_2_ fold change (FC) ≥ 1) (Figure 2a). Of these, 54 genes were shared between the Ob vs. C and Ob + RSV vs. Ob comparisons (Figure 2b), indicating that RSV modulates a subset of obesity-induced gene expression changes.

Hierarchical clustering showed distinct transcriptomic profiles across the three groups. Heatmaps revealed obesity-associated gene alterations (Figure 2c) and their partial normalization in response to RSV (Figure 2d).

### 3.3. Classification of Resveratrol-Regulated Genes

Among the 54 DEGs modulated by RSV in mice with obesity, 55% were protein-coding genes (53.3% downregulated and 46.7% upregulated), whereas a substantial proportion (45%) corresponded to non-coding RNAs. This highlights non-coding transcriptional regulation as a prominent feature of the RSV-responsive hypothalamic transcriptome. The non-coding fraction included antisense RNAs (20.8%, 80% downregulated), long intergenic non-coding RNAs (37.5%, 66.7% downregulated), pseudogenes (20.8%, all downregulated), unprocessed pseudogenes (4.2%, all upregulated), processed transcripts (4.2%, all downregulated), and transcripts to be experimentally confirmed (TEC; 12.5%, all upregulated). Gene classifications and expression data are detailed in Table 1.

Notably, the high proportion of non-coding RNAs among RSV-responsive genes represents a distinctive feature of this transcriptomic response, distinguishing it from profiles dominated by protein-coding genes and highlighting the prominence of non-coding transcripts in the hypothalamic response to RSV under obesogenic conditions.

### 3.4. GO and KEGG Pathway Enrichment Analyses

To further predict the biological effects of RSV in the hypothalamus of mice with obesity, GO and KEGG pathway enrichment analyses were performed using the 54 RSV-responsive DEGs.

**Figure 3:**
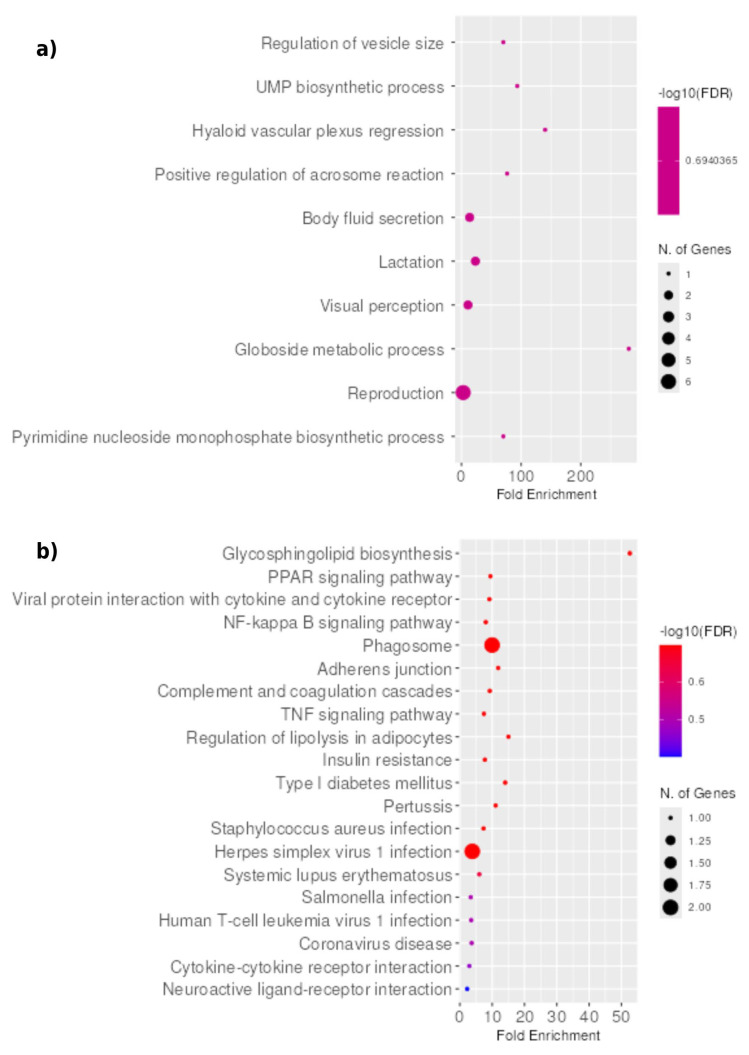
Functional enrichment analysis of biological processes by Gene Ontology (GO) and KEGG pathways associated with RSV-regulated DEGs in the hypothalamus of mice with obesity. (**a**) Enriched GO biological processes. (**b**) Enriched KEGG signaling pathways. The y-axis shows the names of the enriched GO terms or KEGG pathways, while the x-axis represents the enrichment score. The color gradient indicates statistical significance, expressed as −log_10_ of the false discovery rate (FDR), and the size of each bubble corresponds to the number of differentially expressed genes (DEGs) associated with each term. Only terms with an adjusted *p* < 0.05 and |log_2_ fold change| ≥ 1 were included in the analysis.

GO enrichment analysis identified biological processes including globoside metabolism, UMP biosynthesis, regression of the hyaloid vascular plexus, regulation of acrosome reaction and regulation of vesicle size (Figure 3a). Although diverse, these processes converge on lipid handling and vesicular dynamics, both of which are relevant to hypothalamic metabolic regulation.

KEGG pathway analysis revealed enrichment in pathways related to lipid metabolism, inflammation, and insulin resistance, including regulation of lipolysis in adipocytes, PPAR signaling, TNF signaling, NF-κB signaling, and complement cascades (Figure 3b). These enriched pathways support the prediction of RSV-mediated modulation of inflammatory and metabolic signaling within the hypothalamus.

### 3.5. Key Obesity-Related Genes Modulated by Resveratrol

RSV significantly modulated the expression of nine genes that were dysregulated by HFD-induced obesity. Of these, seven genes were upregulated in obesity and downregulated following RSV treatment, including *Aquaporin 7* (*Aqp7*), *Chemokine* (*C-C motif*) *ligand 27A* (*Ccl27a*), *Lymphotoxin alpha* (*Lta*), *Rab interacting lysosomal protein* (*Rilp*), *Mannose-6-phosphate receptor pseudogene* (*M6pr-ps*), *Complement component 1*, *r subcomponent A* (*C1ra*), and *Snail family zinc finger 1* (*Snail1*), and two genes were downregulated in obesity and upregulated by RSV, including *Globoside alpha-1*,*3-N-acetylgalactosaminyltransferase 1* (*Gbgt1*) and *Peroxisome proliferator-activated receptor gamma coactivator 1 beta* (*Ppargc1b*). Table 2 presents the RSV-regulated DEGs in the context of obesity, along with the KEGG signaling pathways influenced by these genes.

**Figure 4:**
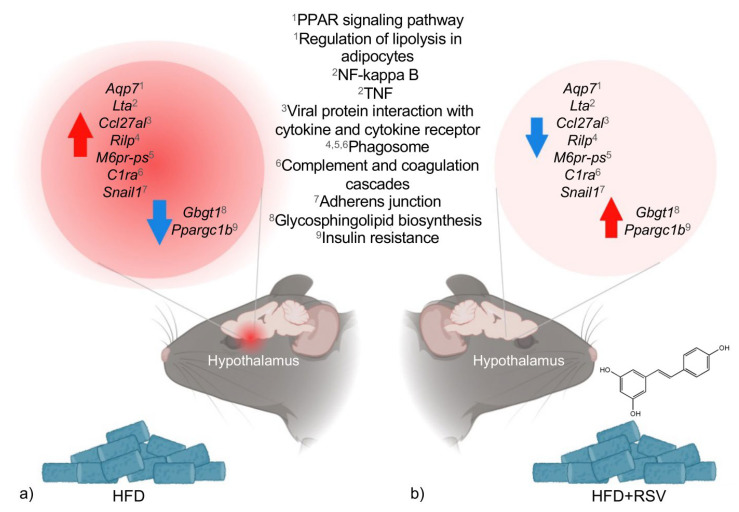
KEGG signaling pathways associated with gene expression changes induced by HFD and modulated by RSV in the hypothalamus. (**a**) DEGs and associated KEGG pathways identified in the hypothalamus of HFD-fed mice compared with controls. (**b**) DEGs and KEGG pathways modulated by RSV treatment in HFD-induced mice with obesity. Red upward arrows indicate upregulated genes, whereas blue downward arrows indicate downregulated genes in each experimental comparison. Superscript numbers next to each gene indicate the corresponding KEGG pathways (numbered in the central panel) to which each gene is functionally annotated.

These genes are implicated in processes such as inflammation, immune response, lipid metabolism, and insulin resistance, which are mechanisms predicted to be reprogrammed by RSV. Figure 4 illustrates the genes and biological functions regulated by RSV.

## 4. Discussion

In this study, we examined the impact of RSV treatment on the hypothalamic transcriptome of mice with HFD-induced obesity. Although several studies have reported that higher doses of RSV reduce body weight in animals fed an HFD [43,44], at the dose used in the present study, RSV administration did not significantly reduce body weight (Figure 1). Nevertheless, RSV exerted a marked effect on transcriptomic modulation. Our transcriptomic analyses revealed that RSV modulates a specific subset of genes involved in inflammation, immune response, lipid metabolism, and energy homeostasis in the hypothalamus. These findings support the hypothesis that RSV can exert neuroprotective and metabolic effects independent of weight loss, consistent with previous reports in rodent models of obesity [27].

Interestingly, a significant proportion (45%) of RSV-modulated transcripts were non-coding RNAs, including lncRNAs, antisense RNAs, and pseudogenes. Emerging evidence indicates that lncRNAs are key regulators of inflammatory signaling pathways and metabolic control in the context of obesity and insulin resistance [45]. Moreover, RSV has been shown to modulate the expression of hundreds of lncRNAs, reversing dysregulated transcriptomes in insulin-resistant skeletal muscle; for instance, the lncRNA NONMMUT044897.2 was downregulated upon RSV treatment, leading to improved insulin sensitivity via the SOCS1 pathway [46]. Although the functions of most RSV-modulated non-coding RNAs in the hypothalamus remain to be explored, their regulation suggests a potential epigenetic mechanism through which RSV exerts central metabolic and anti-inflammatory effects.

Consistent with the literature, our results suggest that an HFD promotes lipid accumulation in visceral adipose tissue, which in turn triggers systemic low-grade inflammation characterized by the release of pro-inflammatory cytokines, free fatty acids, and adipokines [6,12,13,47]. These factors impair hypothalamic function and disrupt circuits regulating energy balance and appetite, leading to a pro-inflammatory transcriptional profile in the hypothalamus [12,13]. In our model, an HFD altered hypothalamic gene expression, while RSV treatment counteracted these changes (Figure 2). KEGG analysis (Figure 3b) revealed that RSV-enriched pathways included NF-κB and TNF signaling, driven by modulation of genes such as *Lta* and *Ccl27a*, both of which are implicated in neuroinflammation and metabolic dysfunction [48,49,50].

*Lta*, encoding lymphotoxin-α, is upregulated in obesity and contributes to inflammation, insulin resistance, and weight gain, while its inhibition confers metabolic protection [48,49]. Similarly, *Ccl27a*, a chemokine involved in T-cell recruitment and neuroinflammatory signaling [50], was downregulated by RSV in our study. Elevated expression of chemokines, including *Ccl2*, *Ccl4*, and *Ccl27a*, has been reported in humans and murine specimens with obesity, correlating with inflammation and insulin resistance, and these expression levels are reduced following interventions such as bariatric surgery [50,51]. In the CNS, *Ccl27* promotes T-cell chemotaxis and plays a role in hypothalamic inflammation [47]. Furthermore, HFD-induced microglial activation and chemokine overexpression have been associated with reduced energy expenditure and obesity progression [14].

The RSV-mediated downregulation of *Lta* and *Ccl27a* observed here may thus reflect a central anti-inflammatory mechanism with potential metabolic benefits. These findings support the proposed therapeutic value of targeting the chemokine-receptor signaling axis to mitigate neuroinflammation and metabolic dysfunction in obesity and related diseases [52,53,54].

RSV also modulated genes involved in lipid and glycerol metabolism. Gbgt1, which participates in glycosphingolipid biosynthesis [55], was upregulated by RSV. Altered glycosphingolipid metabolism has been linked to disrupted lipid handling in metabolic tissues [56], suggesting a potential protective role for RSV through modulation of membrane lipid composition.

*Aqp7*, an aquaglyceroporin involved in glycerol transport, was upregulated in obesity, likely to reflect increased glycerol availability due to HFD-induced lipolysis [57]. RSV treatment reduced *Aqp7* expression, which may be metabolically beneficial, as excessive glycerol influx can contribute to lipotoxicity, oxidative stress, and impaired insulin signaling in the hypothalamus [58]. These findings suggest that RSV-mediated downregulation of *Aqp7* may help protect hypothalamic function under obesogenic conditions.

RSV also restored the expression of *Ppargc1b*, a key regulator of mitochondrial function and energy metabolism that was reduced in obesity. The upregulation of *Ppargc1b* is consistent with studies demonstrating its protective effects against obesity-induced inflammation, insulin resistance, and mitochondrial dysfunction [3,59]. Knockout studies have linked *Ppargc1b* deficiency to increased fat accumulation, systemic inflammation, and impaired glucose metabolism, whereas its overexpression enhances fatty acid oxidation and energy expenditure, thereby conferring resistance to diet-induced obesity [60,61,62]. These findings suggest that RSV may exert protective metabolic effects, at least in part through *Ppargc1b*-mediated pathways.

*In addition*, *RSV normalized the expression of Snail1*, *a gene associated with adiposity and metabolic dysfunction* [63]. Given that *Snail1* deletion leads to reduced adipocyte size and fat mass, its downregulation by RSV suggests potential anti-obesogenic effects. Furthermore, RSV suppressed obesity-induced overexpression of *Rilp*, *M6pr-ps*, and *C1ra*, genes involved in phagosome activation, complement signaling, and coagulation. These pathways are linked to microglial overactivation and excessive phagocytosis, processes associated with neurodegeneration [64,65,66,67]. By dampening these signals, RSV may confer neuroprotective effects by limiting complement-mediated synaptic loss, a phenomenon implicated in Alzheimer’s disease [66,67].

To our knowledge, this is the first study to investigate the impact of RSV on the hypothalamic transcriptome in an HFD-induced obesity model. The integration of RNA-seq and pathway analyses provides novel insight into predictive molecular targets and regulatory networks through which RSV may exert central effects. These findings highlight the potential of RSV as a modulator of hypothalamic inflammation and metabolic dysfunction, although functional validation is still required. The selected dose has previously been shown to exert metabolic effects in HFD-fed mice, supporting its suitability for exploratory transcriptomic analysis [41]. A limitation of this study is the use of a single RSV dose; therefore, dose-dependent transcriptomic effects were not assessed. Notably, despite using a relatively low dose (2.25 mg/kg), we detected significant modulation of hypothalamic gene expression. In contrast, other studies have required higher RSV doses to demonstrate overt neuroprotective and anti-inflammatory effects [68,69]. These findings suggest that transcriptomic changes may be detectable at lower doses, even in the absence of pronounced systemic or weight-related effects.

Future studies should evaluate how these transcriptomic signatures translate into changes in hypothalamic neuronal activity, systemic metabolism, and behavior. The pronounced modulation of non-coding RNAs further emphasizes the need for mechanistic studies assessing their roles in central metabolic regulation. Moreover, given the absence of significant weight loss in our model, it will be essential to determine the dose dependency, long-term efficacy, and translational feasibility of RSV, considering its limited bioavailability and complex pharmacokinetics.

In summary, RSV modulated the expression of hypothalamic genes dysregulated by obesity, including *Aqp7*, *Ccl27a*, *Lta*, *Rilp*, *M6pr-ps*, *C1ra*, *Snail1*, *Gbgt1*, and *Ppargc1b*. These transcriptional changes involve pathways associated with inflammation, lipid metabolism, immune activation, and mitochondrial function. Notably, RSV’s central effects appear to occur at least in part independently of body weight changes and involve coordinated regulation of both coding and non-coding RNAs. Together, these findings provide predictive insights into the molecular mechanisms through which RSV may ameliorate hypothalamic dysfunction in obesity.

## Figures and Tables

**Table 1:** Differentially expressed genes (DEGs) by biotype in the hypothalamus of obese mice and RSV-treated obese mice.

Gene Biotype	Ensembl Gene ID	KEGG Symbol	Gene Name	Log_2_ FC Ob vs. C	Log_2_ FC Ob + RSV vs. Ob
Antisense	ENSMUSG00000085323	*9130410C08Rik*	*RIKEN cDNA 9130410C08 gene*	3.88	−6.26
	ENSMUSG00000098720	*Gm27239*	*Predicted gene 27239*	1.77	−2.29
	ENSMUSG00000087004	*Gm14154*	*Predicted gene 14154*	4.40	−4.46
	ENSMUSG00000092130	*D030025P21Rik*	*RIKEN cDNA D030025P21 gene*	5.61	−5.64
	ENSMUSG00000085134	*Gm11659*	*Predicted gene 11659*	−3.99	4.25
LincRNA	ENSMUSG00000116256	*Gm32405*	*Predicted gene 32405*	5.36	−5.39
	ENSMUSG00000109149	*Gm19656*	*Predicted gene 19656*	3.45	−3.58
	ENSMUSG00000086952	*Gm12596*	*Predicted gene 12596*	5.42	−5.45
	ENSMUSG00000111399	*Gm47328*	*Predicted gene 47328*	5.86	−5.88
	ENSMUSG00000097819	*Gm26813*	*Predicted gene 26813*	5.14	−5.17
	ENSMUSG00000111970	*Gm47715*	*Predicted gene 47715*	5.42	−5.45
	ENSMUSG00000086464	*Gm15835*	*Predicted gene 15835*	−5.20	4.57
	ENSMUSG00000114148	*Gm47701*	*Predicted gene 47701*	−1.99	2.04
	ENSMUSG00000115545	*Gm48908*	*Predicted gene 48908*	−5.75	6.26
Processed pseudogene	ENSMUSG00000112048	*Gm48750*	*Predicted gene 48750*	5.28	−5.31
	ENSMUSG00000080849	*Gm15702*	*Predicted gene 15702*	4.35	−5.12
	ENSMUSG00000115593	*Gm4824*	*Predicted gene 4824*	4.48	−3.62
	ENSMUSG00000083305	*Gm13315*	*Predicted gene 13315*	4.03	−6.36
	ENSMUSG00000080832	*M6pr-ps*	*Mannose-6-phosphate receptor pseudogene*	2.65	−3.48
UP	ENSMUSG00000080888	*Gm14387*	*Predicted gene 14387*	−4.53	5.30
PT	ENSMUSG00000053749	*Gm9920*	*Predicted gene 9920*	5.44	−5.47
TEC	ENSMUSG00000109679	*Gm45342*	*Predicted gene 45342*	−5.38	5.50
	ENSMUSG00000111107	*Gm48737*	*Predicted gene 48737*	−5.46	5.24
	ENSMUSG00000107647	*Gm44445*	*Predicted gene 44445*	−4.57	4.98
Protein coding	ENSMUSG00000060981	*Hist1h4h*	*Histone cluster 1 H4h*	1.42	−1.39
	ENSMUSG00000025401	*Myo1a*	*Myosin IA*	1.13	−1.23
	ENSMUSG00000021345	*Prl8a6*	*Prolactin family 8*, *subfamily a*, *member 6*	5.27	−5.29
	ENSMUSG00000024402	*Lta*	*Lymphotoxin A*	1.96	−2.64
	ENSMUSG00000054626	*Xlr*	*X-linked lymphocyte-regulated*	3.54	−3.63
	ENSMUSG00000095247	*Ccl27a*	*Chemokine* (*C-C motif*) *ligand 27A*	3.92	−4.90
	ENSMUSG00000024175	*Tekt4*	*Tektin 4*	1.21	−1.46
	ENSMUSG00000053877	*Srcap*	*Snf2-related CREBBP activator protein*	−1.19	1.50
	ENSMUSG00000027801	*Tm4sf4*	*Transmembrane 4 superfamily member 4*	3.25	−4.97
	ENSMUSG00000023153	*Tmem52*	*Transmembrane protein 52*	1.44	−1.50
	ENSMUSG00000021506	*Pitx1*	*Paired-like homeodomain transcription factor 1*	4.72	−3.36
	ENSMUSG00000042821	*Snail1*	*Snail family zinc finger 1*	2.66	−2.59
	ENSMUSG00000028427	*Aqp7*	*Aquaporin 7*	5.37	−5.40
	ENSMUSG00000055172	*C1ra*	*Complement component 1*, *r subcomponent A*	3.41	−2.81
	ENSMUSG00000044814	*Olfr543*	*Olfactory receptor 543*	−3.02	3.24
	ENSMUSG00000073514	*Dok6*	*Docking protein 6*	−1.35	1.53
	ENSMUSG00000090053	*Palm2*	*Paralemmin 2*	−1.48	1.73
	ENSMUSG00000026829	*Gbgt1*	*Globoside alpha-1*,*3-N-acetylgalactosaminyltransferase 1*	−2.70	2.97
	ENSMUSG00000033871	*Ppargc1b*	*Peroxisome proliferative activated receptor*, *gamma*, *coactivator 1 beta*	−1.93	1.35
	ENSMUSG00000025776	*Crispld1*	*Cysteine-rich secretory protein LCCL domain containing 1*	−1.23	2.17
	ENSMUSG00000075596	*B130006D01Rik*	*RIKEN cDNA B130006D01 gene*	−5.12	5.76
	ENSMUSG00000038195	*Rilp*	*Rab interacting lysosomal protein*	1.01	−2.13
	ENSMUSG00000043972	*Opn5*	*Opsin 5*	3.27	−5.98
	ENSMUSG00000000263	*Glra1*	*Glycine receptor*, *alpha 1 subunit*	−2.41	2.82
	ENSMUSG00000038048	*Cntnap5c*	*Contactin associated protein-like 5C*	−2.40	2.62
	ENSMUSG00000047773	*Ankfn1*	*Ankyrin-repeat and fibronectin type III domain containing 1*	−2.00	1.45
	ENSMUSG00000073016	*Uprt*	*Uracil phosphoribosyltransferase*	−2.81	2.31
	ENSMUSG00000068130	*Zfp442*	*Zinc finger protein 442*	−2.42	2.96
	ENSMUSG00000097271	*Gm9903*	*Predicted gene 9903*	−4.38	5.18
	ENSMUSG00000096768	*Gm47283*	*Predicted gene*, *47283*	1.01	−1.05

Note: log_2_FC: Log_2_ fold change in gene expression. Only genes showing differential expression between groups with an adjusted *p* < 0.05 and log_2_ fold change ≥ 1 are shown. Biotypes: UP, unprocessed pseudogene; PT, processed transcript; TEC, to be experimentally confirmed. Groups: Ob, obese; C, control; Ob + RSV, obese treated with resveratrol.

**Table 2:** Genes regulated by RSV in the hypothalamus of mice with obesity and their impact on signaling pathways.

KEGG Pathway	KEGG Symbol	Gene Name	Log_2_ FC Ob vs. C	Log_2_ FC Ob + RSV vs. Ob
Glycosphingolipid biosynthesis	*Gbgt1*	*Globoside alpha-1*,*3-N-acetylgalactosaminy* *ltransferase 1*	↓ −2.70	↑ 2.97
PPAR signaling pathway	*Aqp7*	*Aquaporin 7*	↑ 5.37	↓ −5.40
Regulation of lipolysis in adipocytes				
Viral protein interaction with cytokine and cytokine receptor	*Ccl27a*	*Chemokin e* (*C-C motif*) *ligand 27A*	↑ 3.92	↓ −4.90
NF-κB	*Lta*	*Lymphotoxin alpha* (*TNF superfamily*, *member 1*)	↑ 1.96	↓ −2.64
TNF				
Phagosome	*Rilp*	*Rab interacting lysosomal protein*	↑ 1.01	↓ −2.13
	*M6pr-ps*	*Mannose-6-phosphate receptor*, *pseudogene*	↑ 2.65	↓ −3.48
	*C1ra*	*Complement component 1*, *r subcomponent A*	↑ 3.41	↓ −2.81
Complement and coagulation cascades	*C1ra*	*Complement component 1*, *r subcomponent A*	↑ 3.41	↓ −2.81
Adherens junction	*Snail1*	*Snail family zinc finger 1*	↑ 2.66	↓ −2.59
Insulin resistance	*Ppargc1b*	*Peroxisome proliferative activated receptor*, *gamma*, *coactivator 1 beta*	↓ −1.46	↑ 1.28

Note: log_2_FC: Log_2_ fold change in gene expression. Only genes showing differential expression between groups with an adjusted *p* < 0.05 and |log_2_ fold change| ≥ 1 are shown. Up: ↑, down: ↓. Groups: Ob, obesity; C, control; Ob + RSV, obesity treated with resveratrol.

## Data Availability

The original contributions presented in this study are included in the article/Supplementary Materials. Further inquiries can be directed to the corresponding authors.

## Supplementary Materials

The following supporting information can be downloaded at: https://www.mdpi.com/article/10.3390/genes17030297/s1, Table S1: Summary of hypothalamus sequencing data.
