# Supplementary material for: A Predictive Transcriptomic Approach to the Resveratrol-Mediated Reversal of Hypothalamic Alterations in a Mouse Model of Obesity
Source: Genes (Basel). 2026 Feb 28;17(3):297. doi: 10.3390/genes17030297 (PMC13025380; doi:10.3390/genes17030297)
Supplement: Supplementary file 1 [file genes-17-00297-s001.zip › genes-4151654-supplementary.pdf]

Supplementary Table S1. Summary of hypothalamus sequencing data

Sample	Raw reads	Clean reads	Raw bases (G)	Clean bases (G)	Error rate (%)	Clean bases Q20 (%)	Clean bases Q30 (%)	GC (%)
C (1)	61554978	59407570	9.23	8.91	0.03	97.70	93.78	49.15
C (2)	43519550	42360976	6.53	6.35	0.03	97.20	92.77	50.36
C (3)	54859116	51295150	8.23	7.69	0.03	97.24	92.59	47.72
Ob (1)	48472094	47244058	7.27	7.09	0.03	97.25	93.28	49.72
Ob (2)	46032506	45039342	6.90	6.76	0.03	97.40	92.94	51.79
Ob (3)	42785106	45716864	6.42	6.15	0.03	97.02	92.48	47.66
Ob+RSV (1)	62686402	60691422	9.40	9.10	0.03	97.63	93.82	50.04
Ob+RSV (2)	59859876	57501598	8.98	8.63	0.03	96.70	91.77	49.70
Ob+RSV (3)	47723186	45716864	7.16	6.86	0.03	97.04	92.43	49.96

Note: Q20, Q30: (Base count of Phred value > 20 or 30) / (Total base count). G/C: Guanine/Cytosine. Groups: Ob, obese; C, control; Ob+RSV, obese treated with resveratrol.
